# Cherokee Medical Society

**Published:** 1890-06

**Authors:** 


### CHEROKEE COUNTY MEDICAL SOCIETY.

Regular session, Alto April, 1890. Dr. B. F. Brittain, of Jacksonville, President, Dr. J. K. Frazer, of Rusk, Secretary. After prayer, the Committee on Collective Investigation of Disease reported through Chairman J. H. Evans. The subject investigated was Erysipelas. The answers received showed a pretty equal division of opinion as to its ætiology, some claiming germ origin, some that it is a "blood poison," or "constitutional trouble."

Dr. J. N. B. Guinn, of Alto, opened discussion. He thought the cause atmospheric, and the disease may develop from local or constitutional causes, the antecedents not inflammatory in most cases, but simply febrile, the disease occurring mostly in spring and autumn. The constitutional disorder is secondary and of an inflammatory character; is not contagious but infectious.

Dr. Kirksey agreed in the "atmospheric" causation, and its action is mostly confined in the skin.

Dr. Nelson thought erysipelas purely a local trouble. Dr. McCord ascribed the cause to the presence of "germs," and the disease is both contagious and infectious. Dr. Guinn of Rusk, also believed in the germ causation, and thought the invasion may be resisted by nerve force. Drs. Hunter of Larissa, and Brittain of Jacksonville, joined in the discussion, expressing the view that the disease is a constitutional one, in which opinion Dr. Frazer concurred. Dr. Evans held to the germ theory. Dr. Frazer thought some persons are more susceptible to attack than others; did not know what caused the blood poison; thought it infectious, and contagious only where the skin was abraded or a wound exists.

As to the treatment, there was a pretty general unanimity of opinion; quinine and iron; iron internally and externally.

The subject selected for investigation and report at the next meeting was the vexed and much discussed one of Puerperal Eclampsia.

Dr. A. H. McCord read a paper on post-partem hemorrhage. Dr. Guinn, of Alto, read one on same subject; both were highly interesting and instructive. Next, a paper on typho-malarial fever by Dr. Guinn; one on general influenza, by Dr. Brittain. The Doctor gave a history of the disease from the well known attack of President Tyler to date, and thought the modern "grippe" was the same disease under another name.

For the regular essays at next meeting were chosen the following subjects: "The necessity for each family to have a family physician,"—Dr. McCord essayist. "What is the physical effect of Atropia"—Dr. W. W. Pugh. "Anæsthetics, their range of usefulness, and the safest one to use;"—Dr. H. V. Collins; and "Cholera Infantum," by Dr. A. W. Barten.

The courteous Secretary, Dr. Frazer, in transmitting a synopsis of the proceedings says: "In many respects this was one of the best meetings we have had; the members present manifested a lively fraternal interest, which seemed to give a new impetus to their determination to make a success of the Association; the only regret was, that all the members were not present. We were honored with the presence of quite a number, including some prominent citizens of Alto. The thanks of the meeting were voted the good citizens, the local physicians and the Grange, for their kindness. The meeting adjourned to meet at New Birmingham on the 2nd Thursday in July, at 10 a. m."

"We are trying to teach the laity the objects and importance of these meetings; and they take great interest in our sittings."

---

LAREDO, THE GATE TO MEXICO.—Some of the physicians at Laredo are considerably exercised because the State Health Officer has not appointed a qnarantine officer at that post. Only a guard is kept there, and the JOURNAL is informed that the guard will stop a case of small-pox or other contagious disease and telephone for a doctor; no doctor will answer the call because there is no provision for his fee; and hence, it is represented to the JOURNAL that it is not unlikely that, as there is practically no restrictions on the nomadic Mexicans—an epidemic of small-pox may be lighted up at any time. Dr. Rutherford, we learn, has been appealed to, but so far has not redressed the grievance. He should look into it. Is the lesson so soon forgotten, taught by an outbrreak of yellow fever a few years ago, and which cost $20,000 to suppress? an outbreak in consequence of withdrawing an officer at one point in the interest of economy?

